# From Sick Bed to Death Bed? Patient Composition and Mortality in the Amsterdam Binnengasthuis, 1856–1896

**DOI:** 10.1093/shm/hkaf046

**Published:** 2025-07-04

**Authors:** Nadeche Diepgrond, Tim Riswick

**Affiliations:** Radboud Group for Historical Demography and Family History, Radboud University, Nijmegen, The Netherlands; Radboud Group for Historical Demography and Family History, Radboud University, Nijmegen, The Netherlands

**Keywords:** hospitals, mortality, patients, Amsterdam, nineteenth century

## Abstract

Hospitals played a central role in the nineteenth century, as these institutions were the so-called gateways to death or places of healing. Who was admitted and if there is inequality in who died is, however, often understudied. Our study examines the development and mortality risks of the patient population in the Binnengasthuis in Amsterdam over time by analysing detailed patient records of people admitted to the hospital in the period 1856-1896. Our results demonstrate that mortality was not extremely high and depended on the admission policy, the composition of the patient populations was very diverse, and that mortality risks were mainly determined by the disease, year, age, and marital status of the admitted patients. This indicates that a diverse population could get a sick bed, for most it would not become their death bed, and that inequality in mortality risks within the hospital based on socioeconomic status or religion was limited.

Hospitals today are considered to be at the centre of modern medicine. It is where the sick and injured are diagnosed, treated and cured, resulting in increased chances of survival. Yet, when the first institutions resembling our modern hospitals developed during the eighteenth and nineteenth centuries, they had a terrible reputation. Accounts of patients themselves, but also from the literature and later scholarship about medical practices and hospital services, suggest that hospitals had high mortality rates and offered few effective treatments. Only at the end of the nineteenth and beginning of the twentieth century when medical science developed and modern medical practices such as anaesthesia and a(nti)sepsis were adopted did the image of hospitals become more positive. As a result, many scholars have argued that hospitals transformed from so-called ‘gateways to death’, serving almost exclusively the elderly, the poor and others from lower social strata, to true medical centres attracting and supporting an ever-growing part of society.^1^

Similar developments were advocated for the *Binnengasthuis*, a public hospital located in the centre of Amsterdam. The ‘excessive and alarming’ institutional mortality rates, low standards of hospital care, the deplorable state of the hospital buildings and overall unsanitary conditions were the main reasons for the poor reputation and the stigmatisation of the institution.^2^ As a result, the sick and wounded were afraid of hospitalisation. A phenomenon commonly known as *gasthuisvrees* (fear of the hospital) at that time.^3^ Presumably, only the ‘poor’, ‘homeless’ and ‘underprivileged’ sought refuge in this institution. After all, the Binnengasthuis was considered, in the words of the Amsterdam doctor Nieuwenhuys who visited the hospital in 1820, ‘an abode of the unfortunate’.^4^ In the course of the nineteenth century, however, the Binnengasthuis gradually transformed into a true medical centre. In this period, generally known as the medicalisation of hospitals, ‘charity gave way to medicine, care to cure, stigma to pride, the mortuary to the recovery room and the poor to the middleclass’.^5^ Eventually, the so-called *gasthuisvrees* changed to a preference of being admitted to the hospital. Patients were more often admitted on medical grounds, as opposed to social indications, and thus even the sick and wounded among the higher ranks of society found their way to the Binnengasthuis.^6^

In this study, we investigate the development of the Amsterdam Binnengasthuis and its patients in the second half of the nineteenth century. In addition to being one of the largest public urban hospitals in Europe at the time, it also offers unique information because of surviving records on patients that include a wide variety of personal details, the duration of their stay, the identified disease (diagnosis), and the result of treatment (e.g. death or departure). We analyse 13,221 patients who were admitted to the hospital in 1856, 1876 and 1896. This makes it possible to go beyond previous studies and to focus on the patient population. The first aim of our study is to examine how institutional mortality changed over time: Did the Binnengasthuis actually transform into a ‘place of healing’ in the second half of the nineteenth century or was it still a gateway to death where most patients did not survive? And were causes of death of patients changing over time? The second aim is to study the patient composition: Did the social, demographic and religious profiles of those being admitted to the hospital differ over time? The third aim of our study is to examine the background of patients and mortality rates: Did certain patients have higher or lower mortality chances? If so, is there any kind of inequality in care that can be observed within the hospital?

In short, while this study builds on previous research with a narrow focus on the architecture of the hospital, renowned medical staff and some institutional components, we mainly focus on the patients themselves. This enables us to ask new questions about similarities and differences over time and between social groups in the morbidity and mortality patterns of the understudied hospital patients. In the following section, the literature on nineteenth century European hospital care is discussed, including the limited number of studies that explicitly examine patient populations. Next, background information on the Amsterdam Binnengasthuis is provided by placing it within the (inter)national context. The findings are presented after describing the sources, data and methods used in this study. The article concludes with a discussion on how our results of the Amsterdam Binnengasthuis patient population increase our understanding of the history of European hospitals, and how it offers suggestions for future research.

## Background

Throughout the past century the history of hospitals has been dominated by a variety of opposing views regarding the impact of hospitals on public health and patients residing within these institutions. This is partly the result of the great diversity of hospitals, as some originated from Mediaeval elderly care institutions, while others emerged within the context of medical research and education at universities. Nevertheless, it was argued until the 1950s that the remarkable expansion of hospitals in the eighteenth and nineteenth centuries—both in size and numbers—and medical developments, such as the improvement of diagnostics, the first inoculations (smallpox) and the implementation of anaesthesia and a(nti)sepsis demonstrated that improved medical care made a significant contribution to the decline in mortality and consequent population growth.^7^

The most important criticism of the supposed contribution of hospitals to reducing mortality came from McKeown.^8^ He developed a more pessimistic view of eighteenth and nineteenth century hospitals by stating that the aforementioned improvements in medical care and knowledge had no demonstrable effects on public health before the 1940s. Although hospitals and their bed capacity increased, the results of hospital treatment were, according to McKeown, ‘far from reassuring’^9^ and hospitals could best be characterised as ‘gateways to death’. This view, being part of the ‘McKeown thesis’ was, and still is, one of the most influential and ground-breaking positions within this historiographical debate about medical care and public health. Adopting McKeown’s views, Deane argued that hospitals at that time were contributing to the further spread of diseases, rather than containing them, and Mathias argued that hospitals were ‘hot-beds of infection’ and that there was no evidence that these institutions in any way improved the chances of survival and life expectancy of their patients.^10^ This eventually led McKeown to conclude that hospitals at the time made no significant contribution to public health. Even more so, he suggested that ‘the chief indictment of hospital work at this period is not that it did no good, but that it positively did harm’.^11^ In line with this pessimistic view of eighteenth- and nineteenth-century hospitals, the image of the hospital patient as indigent and coming from the lowest social classes and margins of society was clearly present. Although McKeown and his followers did not pay much attention to the hospital patients themselves in their research, the early modern hospital was for a long time stigmatised by many as a locus of pauperism.^12^ It gave no hope to their patients and was thus avoided by the majority who had the possibility to be cared for at home. It was believed that it was only used by an unfortunate minority as a last resort.^13^

In the 1970s a ‘new’ hospital historiography emerged, in which the horrifying reputation with which the early modern hospitals had been endowed was reconsidered by questioning that if the hospital was in fact a so-called ‘gateway to death’, why were people willing to enter them at all? Sigsworth, was among the first to challenge McKeown’s hypothesis in 1972. Based on his findings that the percentage of patients who died in the English York County Hospital rarely exceeded 10 per cent of the total number of admitted patients in the eighteenth and nineteenth century, which he considered to be acceptable.^14^ In the following decades other historians had similar observations based on extensive research on records of admission and dismissal of patients and annual statistics of institutional mortality in various hospitals in the United Kingdom and Sweden (Table 1). In general, they presented a more nuanced view of these medical institutions, and concluded that institutional mortality was not as excessive as McKeown and others presumed. While some hospitals lived up to their bad reputation—for instance at least one in ten patients did not survive hospitalisation in the Manchester Royal Infirmary in the third quarter of the nineteenth century—in most hospitals mortality was remarkably low (between 2 and 6 per cent). Woodward concluded that institutional mortality varied from one hospital to another, due to differences in geographic location, size and hospital admission policy.^15^ In addition, Brändstrom and Broström perceived considerable differences between patients regarding their diagnosis: 21.4 per cent of patients with infectious diseases died during hospitalisation, whereas 2.3 per cent with venereal diseases did not survive hospital treatment.^16^ In conclusion, most hospitals were not gateways to death for all patients during all time periods.^17^

**Table 1.** Mortality in various hospitals in the second half of the nineteenth century

Hospital	Period	Mortality % (range)
County Hospital of Sundsvall	1844–1900	10.3
Bristol Royal Infirmary	1850–1880	5.1–6.5
Worcester General Infirmary	1850–1880	4.1–5.5
Leeds General Infirmary	1850–1880	6.4–7.2
Addenbrooke’s Hospital	1850–1880	5.3–5.6
Leicester Royal Infirmary	1850–1880	4.5–4.6
Norfolk and Norwich Hospital	1850–1880	4.9–5.4
Salisbury General Infirmary	1850–1876	2.3–5.8
Hull Royal Infirmary	1850–1875	4.8–9.2
Manchester Royal Infirmary	1849–1875	9.6–13.4
Liverpool Royal Infirmary	1850–1875	6.1–9.1
Edinburgh Royal Infirmary	1849–1875	9.3–10.9

Mortality (%) = number of deaths in the hospital as a percentage of the number of all admitted patients. Sources: The County Hospital of Sundsvall (Anders Brändström and Göran Broström, ‘Life-Histories for Nineteenth-Century Swedish Hospital Patients: Chances of Survival’, *Journal of Family History,* 1989, 14, 195–209); Bristol Royal Infirmary, Worcester General Infirmary, Leeds General Infirmary, Addenbrooke’s Hospital, Leicester Royal Infirmary, Norfolk and Norwich Hospital (Steven Cherry, ‘The Hospitals and Population Growth: Part 2. The Voluntary General Hospitals, Mortality and Local Populations in the English Provinces in the Eighteenth and Nineteenth Centuries’, *Population Studies*, 1980, 34, 251–265); Salisbury General Infirmary, Hull Royal Infirmary, Manchester Royal Infirmary, Liverpool Royal Infirmary, Edinburgh Royal Infirmary (John Woodward, *To do the sick no harm. A study of the British voluntary hospital system to 1875* (London: Routledge, 1974)).

Next to demonstrating that mortality levels were not as high as expected, the new hospital historiography also highlighted an aspect that had long been neglected and understudied: patients residing within these institutions. Scholarly attention has shifted from the extensively researched medical staff to the unknown patient. For the first time, using the extensive archives of admission and discharge of patients, several historians managed to give hospital patients their own identity and history.^18^ For instance, Imhof concluded that the population of hospitals in Berlin, Kongsberg and Copenhagen not solely consisted of people of the lowest marginal strata, but also the working class and ‘deserving poor’, the latter referring to those considered worthy of help. Only the middle or upper class seemed to be able to avoid hospitalisation at all times, given their preconceived attitudes toward the hospitals.^19^ Brändström and Broström as well as Von Bueltzingsloewen later confirmed these findings, concluding that a wide variety of patients—in terms of sex, age, geographical origin, profession, etc.—were admitted to the hospital in the nineteenth century. However, hospitalisation was not yet widespread among all social classes.^20^ Derosas and Munno, researching the Venetian registers of death, argued that mostly the elderly and poor, who lived in miserable conditions and could not count on any help from their social circles and family network, were hospitalised. Although in the process of medicalisation, the hospital was increasingly preferred to treatment at home, patients ‘[...] resorted to the hospital on the assumption that it gave access to treatments otherwise unavailable or of lesser quality’.^21^

While the aforementioned studies investigating mortality in hospitals in Venice, Sweden, and London have shown that a significant proportion of all deaths in the nineteenth century occurred in hospitals, the sources used only rarely offer sufficient information to examine in-depth who was admitted, and who died in its care. Moreover, for the Netherlands, no single study exists on the historical development of hospitals and patient care from the patient’s viewpoint. Therefore, our study focuses on one of the largest hospitals in the Netherlands at the time, the Binnengasthuis, of which the sources containing this information are available. Because of these detailed sources we can provide an in-depth description of the history of hospital patients and patient care, thereby giving them their own history and placing them firmly within the arena of medical and demographic history. The few earlier studies suggest that next to admission policies, patient composition and mortality risks depended on the historical context in which the hospital was located. We therefore re-examine whether the Amsterdam Binnengasthuis should be considered ‘a gateway to death and abode for the unfortunate’ or if it may actually have been increasingly attracting, supporting and healing an ever-growing part of society over time. In short, by studying the Binnengasthuis as a case study during a time when some of the greatest medical revolutions were taking place, we hope to incorporate the Dutch context into the international debate, as it may offer new perspectives on the history of European hospitals.

## Historical Context

In the past centuries the Amsterdam Binnengasthuis has performed various functions. The ‘Sint Pietersgasthuis’, the predecessor of the Binnengasthuis, was founded in the late Middle Ages by a religious guild and was therefore originally an institution of Christian charity, offering shelter to the chronically ill, travellers and the homeless.^22^ In the seventeenth and eighteenth centuries the institution gradually transformed into a centre of urban healthcare. This development was initiated in 1686 when the mayors of the Amsterdam City Council stated that the Binnengasthuis was exclusively intended for the indigent sick inhabitants of the city, and that foundlings, orphans and disabled soldiers would no longer be admitted.^23^ In the eighteenth century, the Binnengasthuis, like many other large and outdated hospitals in Europe, had to cope with major difficulties; that originated from declining revenues and rising patient numbers, resulting in low standards of hospital care.

In the first half of the nineteenth century, economic conditions in Amsterdam were miserable. Large-scale unemployment, immense poverty, abysmal living conditions—in conjunction with frequent outbreaks of cholera, diphtheria, smallpox, measles and other ‘fevers’—created a particularly unfavourable state of public health.^24^ This resulted in a huge influx of patients, although the financial resources of the institution were limited. At this time, the situation in the Binnengasthuis worsened and eventually became untenable due to high institutional mortality rates, low standards of care and the institution’s poor reputation.^25^ Moreover, the exterior and interior of the hospital buildings remained virtually unchanged for centuries, which led to overcrowding as patient numbers continued to increase. As a result, ‘the bedsteads or cots no longer served two patients, but four or even six’.^26^ Nursing personnel generally worked in the hospital without any form of education. Most stemmed from the lowest social classes and were all subjected to long working hours, low wages and miserable working conditions.^27^ Drunkenness, eating or selling the food intended for patients and even assaults on hospitalised patients were therefore not uncommon. This resulted in an insufficient state of patient care.^28^ According to Hellinga, head of the Binnengasthuis in 1930, the hospitalised sick and injured were ‘at the mercy of heathens’ in the early nineteenth century.^29^ Finally, the almost complete absence of hygienic measures also contributed to the high mortality rates and unfavourable reputation of the Binnengasthuis. The hospital was often described as being extremely filthy. For instance, in the absence of an operating room surgery was performed within the hospital ward in the presence of other patients, and there was only one bathtub, which was rarely used, in the entire hospital.^30^

During the course of the nineteenth century, however, the Binnengasthuis gradually developed into a more modern medical centre. This development started with the establishment of the *Klinische School* (medical school) in the Binnengasthuis in 1828. Thereafter, the hospital was no longer exclusively an institution for caring for and curing patients, but also became an educational centre for medical practitioners in training. The establishment of the medical school within the hospital and the appointment of several professors benefited medical education and research. Another positive consequence was that the fate of the patients improved, as these professors were responsible for those admitted to the hospital and were dedicated to making improvements in the hospital.^31^ For example, professors Tilanus and Suringar, ensured that several large wards were divided up in smaller rooms, the surgical department was expanded with a separate operating room and two isolation rooms, the wooden cots were replaced with iron beds, a separate bathroom was built in each building and the hospital was provided access to clean water.^32^

During the second half of the nineteenth century, the number of admitted patients continued to grow and several developments, such as the continuous advancement in medical science, the professionalisation of the nursing staff and the drastic renovation of the hospital buildings, presumably improved the state of health care. Firstly, prominent medical developments and discoveries found their way to the Binnengasthuis.^33^ In 1847, for instance, percussion and auscultation were implemented in the hospital as new medical examination methods, which improved the accuracy of diagnostics. In the same year anaesthesia was used during surgery for the first time.^34^ Thereafter, in the 1860s, the aseptic method was increasingly used, whereby tools and instruments were disinfected, followed by the introduction of antisepsis—disinfection of the wound surface to prevent inflammation and infection—in 1874.^35^ Secondly, as a consequence of a report in 1882 about the miserable state of patient care, it was decided to reorganise nursing practices in the Binnengasthuis. With the establishment of the Amsterdam branch of *het Witte Kruis*, an association formed in 1875 to promote public health and avert epidemics, it became possible to train nurses by means of theoretical lessons and practical training in the Binnengasthuis. The care for the sick and wounded was henceforth entrusted to qualified personnel.^36^ That the hospital also obtained the status of an academic hospital, is further proof of this. Lastly, in 1867, the city council made a final decision to thoroughly renovate the Binnengasthuis. However, due to an ongoing debate about the costs and appropriate layout of the new Binnengasthuis, the drastic renovation of the hospital actually started much later and was not finished before the turn of the century.^37^ In short, because of the professionalisation of the nursing staff, the implementation of new medical discoveries and renovations, the emphasis in the hospital shifted from care to cure during the nineteenth century.

## Sources, Data and Methods

In contrast to previous research, in which the main focus was primarily on annual statistics regarding—for example—institutional mortality rates and the number of admitted, dismissed and deceased patients, in this study, we aim to gain more insight into the patient composition and survival on an individual level. To examine the hospitalised population, we use the patient records of admission and dismissal of the Binnengasthuis.^38^ To this day, this source has never been the object of systematic analysis, even though these comprehensive records offer a wide range of information on the hospitalised patients in nineteenth-century Amsterdam. These patient records provide various demographic and socioeconomic characteristics of the hospitalised population, such as their age, sex, occupation, religious affiliation, marital status, place of birth and residential address. In addition, the date of admission to the hospital and the date of their departure or death, the duration of hospitalisation and—in later years—their diagnosis were also noted. Furthermore, information on the patients’ families is also registered, such as the number of children and the parents’ names, place of residence and survival status.

The registers of admission to and dismissal from the Binnengasthuis have been drawn up from 1850 until 1899 and cover information on approximately more than 200,000 patients in total, as several thousands of patients were admitted to the hospital each year. In this study, we focus on three sample years: 1856, 1876 and 1896. Each is 20 years apart so we are able to study change over time, while still making full use of the time range that the source is available. It is important to state that not the records of all patients in the sample years are taken as the starting point, but only those who were admitted to the hospital in these years. Thus, patients who are listed in the registers of 1856, 1876 and 1896, but were originally admitted to the hospital in previous years are not included in this study. Moreover, missing information, such as the date of departure or death, of hospitalised patients in the sample years was complemented using the patient records of subsequent years, since a significant proportion of the patients admitted was still hospitalised at the end of the year. The total sample consists of 13,211 patients, whose hospital records were digitised with the help of several citizen scientists from the *Radboud Group Citizen Science for the History of Health*.

There are some important advantages and drawbacks to using this source for our study. Firstly, one of the major advantages is the availability of the primary source over a long period of time, which allows us to examine developments and trends throughout the second half of the nineteenth century. Secondly, the extensive amount of information on the hospitalised patients allows us to examine the characteristics of the patient population in depth. Lastly, we can assume that the patient records are complete. This is evidenced, for example, by the fact that even patients who were hospitalised only briefly (no more than a day) or who were admitted while dying—*‘moribund’*—were listed. In addition, we find similar total numbers of patients in other sources, such as the annual municipal reports of Amsterdam.^39^ The largest drawback of the source is that, no distinction was made in the patient records of the Binnengasthuis as to the condition in which the patients arrived and left the hospital. It thus remains unclear if, for example, their condition improved during their stay or whether they were declared ‘cured’ or ‘incurable’.

Next to the described patients records we incorporate three additional sources in our study: the causes of death registers, the rent values from the land registries and municipal and medical reports. Firstly, the cause of death registers have been digitised in the Amsterdam Cause-of-death Database and include the individual-level causes of death of almost everyone who died in Amsterdam during the period 1854–1926.^40^ Almost all patients who died in the hospital could therefore be linked to the cause of death database (between 80 and 90 per cent) based on the date of death, age, sex and address. An advantage of this database is that individual causes of death are coded using the Historical International Classification of Diseases (ICD10h).^41^ Next, the ICD10h codes are grouped using the classification scheme by Reid et al., in their study of the causes of death in Scotland between 1885 and 1949. This makes it possible to also study how causes of death changed over time, as this information is not included in the patient records.^42^

Secondly, because occupation only offers one kind of indicator for socioeconomic status—and is mostly missing for women and children—the digitised rent values from the land registry (*kadaster*) of 1832 and 1907 were also incorporated. These registries record the amount of rent for almost all addresses in Amsterdam, which can be divided in four groups (quartiles) to obtain an indicator for the estimated wealth of individuals living on a specific address. By connecting the addresses listed in the land registry of 1832 to addresses of the patients in 1856, and the land registry of 1907 to addresses of the patients in 1876 and 1896, we have the opportunity to measure socioeconomic status on an individual level for most patients who entered the Binnengasthuis, next to examining their occupation.^43^

Lastly, aggregated information from reports from the municipality (*Gemeenteverslagen* and *Statistische Jaarboeken Amsterdam*) and reports from medical doctors, such as the Amsterdam physician Israëls, are used. Most importantly, this includes Israëls’s categorisation of all 50 neighbourhoods into ‘poor’, ‘rather poor’, ‘rather wealthy’, and ‘wealthy’ based on his knowledge of the city.^44^

Table 2 shows the descriptive statistics of the full dataset and the variables that are further explored in the result section. For most variables the historical categories in the sources are used, with three exceptions. First, the HISCO classification system is used to classify the occupations in five broad categories. Moreover, in some analyses the category of farmers and fishermen is included in those of the Other/Unknown category because their number was very low. Second, the thirteen different religious groups that were recorded were reclassified for our analyses into larger groups such as Protestants, Catholics, Jews and other religious affiliation. Lastly, to be able to explore the differences within the hospital, the thirteen mentioned wards were reclassified to four: Other (Gynaecology, skin, eye, nerve diseases, syphilis and maternity), Infant and Children (Children, Children Sick, Infants), Bandage (Bandage) and Sick (Sick).

**Table 2.** Descriptive statistics patient records

	Count (*N*)	%
**Sex**		
* Men*	6425	48.6
* Women*	6786	51.4
**Year of admission**		
* 1856*	3365	25.5
* 1876*	3104	23.5
* 1896*	6742	51.0
**Occupational group (male)**		
* Unknown*	6651	50.3
* Elite*	20	0.2
* Lower Middle Class*	481	3.6
* Skilled Workers*	2423	18.3
* Farmers and Fishermen*	20	0.2
* Unskilled Workers*	3616	27.4
**Religion**		
* Protestant*	9429	71.4
* Catholic*	3702	28.0
* Jewish*	30	0.2
* Other*	50	0.4
**Hospital Ward**		
* Other*	3094	23.4
* Infants and Children*	620	4.7
* Bandage*	3737	28.3
* Sick*	5760	43.6
Source: Amsterdam Patient Records Database.		

For the first part of the analysis we use descriptive statistics to analyse institutional mortality of the Binnengasthuis over the entire study period. This makes it possible to compare the mortality in the Binnengasthuis with the results obtained in other studies, and explore in what ways, and why, it differed or not. In addition, the development of causes of death is also analysed by examining what patients died of in the sample years. In the second part descriptive statistics are used to study changes and similarities in patient composition over time. These analyses are enriched by referring to individual patients and by comparing the observed trends with what we know about the general population in Amsterdam.

The third part of our analysis focuses on who died in the hospital by employing univariate Kaplan–Meier survival curves and multivariate Cox proportional hazard models. The Kaplan–Meier survival curves are displayed in Figure 2, in which the *x*-axis shows the time in days and the *y*-axis shows the percentage of patients who have not yet died. In short, the risks of dying are calculated cumulatively over time (in days) up to 100 days after admission.^45^ This is done to obtain a first overview of possible important factors determining mortality chances in the Binnengasthuis. Multivariate event history Cox proportional hazard models are applied to analyse survival by taking into account the timing of an event and control variables in Table 5. In Cox proportional hazard models, relative risks of covariates on the timing of a particular event are calculated in terms of hazard rates. These rates can be transformed to hazard ratios, which are easier to understand. A hazard ratio greater than 1.00 indicates that the covariate is associated with an increased hazard of dying compared to a reference category. A hazard ratio less than 1.00 indicates the opposite.^46^ In our Cox proportional hazard models the event is death, and observation time begins at the moment when a patient enters the hospital and ends when a patient dies before or on day 10. When patients do not die during this observation period, they are censored either after 10 days or at the moment they leave the hospital if this is before day 10.^47^ By doing so, the proportionality assumption is met.^48^

**Table 5.** Cox proportional hazard model survival between 0 and 10 days, 1856–1896

Variable	Exp(coef)	SE	*P*-value
**Period**			
*1856*	Ref.		
*1876*	0.849	0.101	0.107
*1896*	0.694	0.113	0.001**
**Age**			
*0-4*	5.737	0.145	0.000***
*5-13*	1.871	0.161	0.000***
*14-34*	Ref.		
*35-49*	2.656	0.125	0.000***
*50+*	5.221	0.122	0.000***
**Religion**			
*Catholic*	Ref.		
*Protestant Reformed*	1.018	0.099	0.854
*Protestant Luther*	1.023	0.120	0.844
*Other & Unknown*	1.017	0.106	0.869
**Marital status**			
*Married*	Ref.		
*Unmarried*	1.090	0.107	0.421
*Widowed*	1.370	0.108	0.004**
*Other and Unknown*	1.018	0.168	0.917
**HISCLASS**			
*Other and Unknown*	1.421	0.112	0.001**
*Upper middle classes*	1.298	0.191	0.173
*Skilled workers*	1.437	0.111	0.001**
*Unskilled workers*	Ref.		
**Rent value**			
*1*	Ref.		
*2*	1.085	0.096	0.397
*3*	0.695	0.135	0.007**
*4*	0.910	0.162	0.560
*Unknown*	1.058	0.098	0.565
**Israel’s wealth classification**			
*Poor neighbourhood*	Ref.		
*Rather Poor neighbourhood*	1.063	0.085	0.471
*Wealthy neighbourhood*	0.968	0.104	0.754
*Unknown*	0.747	0.139	0.037*

*Source*: Amsterdam Patient Records Database.

*Notes*: N =13.059, Events = 832. This model is stratified for sex because this variable violated the Cox Proportional Hazard model. It enables to still control for sex and gender differences, but has the disadvantage that effects cannot be shown.

## Development of Institutional Mortality

First, it is important to note that a significant proportion of the deceased in Amsterdam (7,39 per cent 1854–1900) died in one of the hospitals in the city, based on calculations of the Amsterdam Cause-of-Death Database. Calculations for this specific case study show that about 1 per cent of the deceased in the sample years 1856 and 1876 and 8 per cent in 1896 died in the Binnengasthuis.

**Fig. 1.**
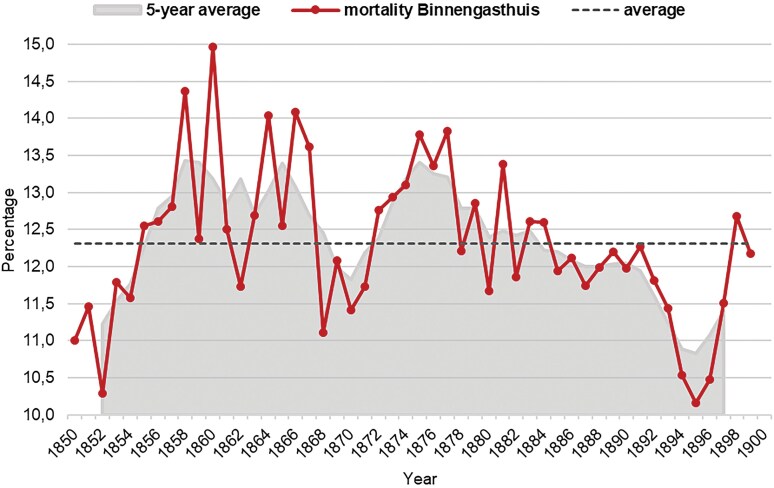
Mortality (%) hospitalised patients Binnengasthuis, 1850–1900. Mortality (%) = number of deaths in the hospital as a percentage of the number of all admitted patients. *Source*: Stadsarchief Amsterdam (Amsterdam City Archives), 5268: Archief van de Gemeenteziekenhuizen, inventory number 2511-2610

The percentage of patients who died in the Amsterdam hospitals fluctuated heavily from one year to another, as Figure 1 shows. The mortality rate ranged from just above 10 per cent (in 1852, 1894–1895) to almost 15 per cent (in 1860). These annual fluctuations were usually a reflection of the state of Amsterdam’s public health in general. When the public health situation was favourable, as it was in 1852 when, according to the municipal annual reports, ‘no diseases of an alarming nature appeared in the city’, this also resulted in relatively low mortality rates in the hospital.^49^ In contrast, in 1877, when the mortality rate in the Binnengasthuis reached a peak again, this year was described in the annual report as: ‘in the bleak spring months mortality was extraordinarily great, to which the diseases of the respiratory organs played a large part’.^50^

Until the mid-1850s, the mortality rate in the hospital was still below the average mortality rate, but soon the mortality rate remained at a relatively high level. With the exception of the period 1868–1871, mortality stayed relatively stable from 1885 onwards, and a clear decline is visible during the 1890s. Yet, during the last observed years mortality was relatively high again. That mortality did not decline earlier in the Binnengasthuis was observed in other European hospitals as well. Cherry and Risse, for instance, observe rising hospital mortality rates due to overcrowding in the course of the nineteenth century.^51^ In the Binnengasthuis the numbers of patients also increased enormously in this period. While in 1870 about 5,000 patients were hospitalised, in 1890 more than 8,000 patients were admitted. The hospital was often not prepared for this large increase in the number of patients, in particular as the extensive renovation and expansion of the building was far from completed during this period. Furthermore, increased medicalisation and advances in institutional health care did not seem to have an effect in improving the fate of patients yet. The most likely explanation is that it took some time before new medical knowledge and insights had a real effect on the quality of hospital care as McKeown and Brown suggested.^52^

In comparison with some other European hospitals in the second half of the nineteenth century, the mortality rate in the Binnengasthuis was relatively high, therefore, as Sigsworth hypothesised, mortality varied considerably from institution to institution. As can be seen in Table 1 (background), even in a ‘favourable’ year, the mortality rate at the Binnengasthuis was still significantly higher than at most other hospitals. The question is, however, to what extent these cases are comparable to the Binnengasthuis? For example, the regional hospital in Sundsvall, Sweden, with an average mortality rate of 10.3 per cent, was a small-scale institution. In the mid-nineteenth century, only 80 patients were admitted annually, whereas in the Binnengasthuis annually 3,000 to 4,000 patients were hospitalised during this period.^53^ Furthermore, most British hospitals, such as the Norwich and Norfolk hospitals, were characterised by strict admission policies, which tended to exclude patients with a high risk of mortality and resulted in a relatively low mortality rate.^54^ In contrast, the Binnengasthuis was an urban institution for public health care with a non-restrictive admission policy, which meant that injured and sick people with a high risk of mortality were regularly admitted, such as infants and patients with infectious diseases. A better comparison can be made with the Manchester Royal Infirmary, where mortality in the third quarter of the nineteenth century ranged from 9.6 per cent to 13.4 per cent. In this hospital, as in the Binnengasthuis, pregnant women and patients with venereal diseases and infectious ‘fevers’ were not excluded from admission. Moreover, about two thousand patients were admitted there around 1850.^55^

Next to how many of the patients died, little research has focussed on what patients died of. In Table 3, the causes of death of patients in the Binnengasthuis in the three studied sample years is shown. It reveals that infectious diseases were the most common cause of death, accounting for over 40 per cent of all patient fatalities. Notably, respiratory diseases, tuberculosis, diphtheria, syphilis and measles were among the infectious diseases that caused the highest number of deaths. This finding aligns with previous research indicating that the hospital’s death rate rose significantly during years when epidemics swept across the city. The proportion of patients dying from ‘other’ infectious diseases decreased markedly in the last quarter of the nineteenth century. This trend suggests that the reduction in contagious infectious diseases, and the absence of major epidemics in Amsterdam during this period, may have contributed to the decline in patient mortality. Despite a decline of certain infectious diseases, mortality rates associated with tuberculosis and other respiratory infections remained high throughout the study period. Medical doctors in, and beyond, the hospital appeared to be making progress in their efforts to combat and cure infectious diseases, such as diphtheria, syphilis and smallpox. However, tuberculosis proved to be a formidable adversary for physicians, as effective treatment methods were not yet available, thus leaving little chance of survival after infection. As a result, patients continued to succumb to tuberculosis and other respiratory diseases at high rates throughout the study period.

**Table 3.** Causes of death of deceased patients in the Binnengasthuis

Year	1856	1876	1896	1856-1896	
Disease	%	%	%	*N*	%
Tuberculosis and other airborne diseases	29.3	35.6	30.3	643	32.1
Other infectious diseases	19.5	5.2	5.5	175	8.7
Circulatory diseases	6.4	9.9	12.0	203	10.1
Diseases of the genitourinary system	5.6	6.6	11.1	170	8.5
Neoplasm	3.2	7.9	8.5	143	7.1
Digestive system diseases	7.3	6.0	6.5	132	6.6
Violence	4.1	7.4	6.4	126	6.3
Nervous system	3.6	4.0	3.5	75	3.7
Old age	2.4	1.1	0.5	23	1.1
Childbirth and Perinatal	1.3	1.2	2.7	39	1.9
Other	7.7	13.8	6.1	159	7.9
Unknown	9.6	1.2	7.0	117	5.8
	*N = 467*	*N = 648*	*N = 910*		*N = 2005* ^a^

^a^ *N* = number of causes of death that could be linked to the patient registers of patients who died. Source: Amsterdam Patient Records Database; Amsterdam Causes-of-Death Database; Stadsarchief Amsterdam (Amsterdam City Archives), 5268: Archief van de Gemeenteziekenhuizen, inventory number 2523–2528, 2563–2572, 2603–2610.

Over time, the incidence of cardiovascular (circulatory) diseases and cancer (neoplasm) grew in prominence as leading causes of death among hospitalised patients. In light of the growing number of deaths caused by cancer, Verdoorn conducted extensive research on the health system in Amsterdam during the nineteenth century. Based on his findings, he noted that while mortality rates from other diseases could not be considered high, the increase in cancer-related deaths (from 1860s onwards) was notable, not only in the Netherlands but also in other parts of Europe.^56^ Several factors contribute to the rise in mortality rates attributed to cancer and cardiovascular disease. Both of these causes of death are frequently categorised as ‘diseases of affluence’ that tend to manifest when a patient has already reached an advanced age. The continual advancements in medical care, coupled with a reduction in mortality rates associated with infectious diseases, including those observed at the Binnengasthuis, have undoubtedly contributed to an increase in the number of patients who died as a result of these chronic conditions. In fact, cardiovascular disease emerged as the primary cause of death in the hospital population over age 50, and, approximately 70 per cent of all cancer-related fatalities occurred among patients in the 50+ age category. Yet, Reid et al. suggested: ‘[...] that the rise in mortality rates due to cardiovascular diseases and cancers […], was not due to a rise in these “degenerative” diseases, but was the result of improving diagnosis’.^57^ In Amsterdam’s Binnengasthuis, one does not seem to exclude the other, and it is quite possible that both explanations contributed to the increase in the number of patients who died of cancer or cardiovascular disease.

In conclusion, our analysis of the causes of death of deceased patients at the Binnengasthuis reveals that the hospital primarily served as a site of death for individuals afflicted with specific deadly illnesses, such as infectious diseases, cancer, and cardiovascular diseases. The high mortality rate observed at the Binnengasthuis was—most likely—largely attributable to the large number of patients and the hospital’s non-restrictive admission policy, which distinguished it from other institutions during the same period. Therefore, it could be argued that the Binnengasthuis remained a gateway to death for patients with specific diseases or injuries until the late nineteenth century, although the mortality rate was predominantly shaped by the patient population it served and not necessarily a result of the bad circumstances and limited medical knowledge within the hospital itself.

## Development of the Patient Population

The study of patients residing within hospitals has been historically neglected, despite the valuable insights that can be gleaned from such research. This section will specifically explore who was admitted to the hospital—were they primarily individuals from lower social strata, the elderly or those lacking family support? In other words, were certain groups overrepresented? Or was hospitalisation widespread across the population, leading to a more diverse patient population? Furthermore, we will analyse changes in the social, demographic, and religious profile of the group of patients over time focussing on sex, age, place of birth and residence, religion, social-economic status and the family network of patients. To do so, we utilise the information in Table 4, which provides an overview of the development of patient composition throughout the period 1856–1896. In short, our analyses demonstrate that the group of admitted patients reflects the general population: patients were men and women, born within or outside the city, from all social classes and came from all religions. Only the very young and old were underrepresented, although they were the most vulnerable in society. Moreover, the Binnengasthuis was not exclusively intended for patients who could not, or to a lesser extent, rely on their relatives for support in times of need, such as ‘singles’ and orphans.

**Table 4.** Descriptive statistics of the variables according to period

	1856	1876	1896
	*N = 3,365*	*N = 3,104*	*N = 6,742*
**Sex**			
* Male*	46.0%	53.8%	47.5%
* Female*	54.0%	46.2%	52.5%
**Age**			
* <1*	0.8%	1.8%	2.7%
* 1–4*	3.2%	4.2%	9.2%
* 5–13*	8.5%	6.8%	11.9%
* 14–19*	10.5%	7.5%	9.7%
* 20–34*	42.6%	36.0%	29.3%
* 35–49*	20.3%	22.8%	18.7%
* 50–64*	9.9%	15.1%	11.9%
* 65–79*	3.9%	5.1%	5.8%
* 80+*	0.2%	0.4%	0.5%
* Unknown*	0.2%	0.2%	0.4%
**Place of birth**			
* Amsterdam*	66.7%	61.1%	57.5%
* Other*	33.1%	38.7%	42.2%
* Unknown*	0.2%	0.3%	0.3%
**Place of residence**			
* Amsterdam*	81.4%	88.1%	92.6%
* Other*	3.2%	7.7%	4.6%
* Unknown*	15.5%	4.2%	2.8%
**Religion**			
* Protestant*	73.0%	70.0%	71.2%
* Catholic*	26.8%	29.6%	27.9%
* Jewish*	-	0.1%	0.4%
* Other*	0.2%	0.3%	0.5%
**Occupational group (male)** ^a^			
* Elite*	0.2%	0.3%	0.5%
* Lower middle class*	6.0%	6.4%	10.0%
* Skilled workers*	45.7%	41.3%	37.8%
* Self-employed farmers and fishermen*	0.3%	0.3%	0.5%
* Unskilled workers and farm workers*	36.3%	41.2%	29.8%
* Occupation unknown*	1.1%	0.9%	3.8%
* No occupation*	10.5%	9.7%	17.8%
**Occupational group (female)** ^a^			
* Elite*	–	–	–
* Lower middle class*	1.1%	1.6%	0.8%
* Skilled workers*	4.1%	6.0%	4.1%
* Self-employed farmers and fishermen*	–	–	–
* Unskilled workers and farm workers*	32.3%	41.1%	27.7%
* Occupation unknown*	0.1%	0.1%	0.1%
* No occupation*	62.4%	51.3%	66.8%
**Rent value address**			
* 1 (lowest)*	31.2%	31.4%	25.4%
* 2*	19.1%	15.8%	23.1%
* 3*	15.0%	5.4%	15.5%
* 4 (highest)*	5.5%	7.2%	8.1%
* 5 (unknown)*	29.2%	40.2%	28.0%
**Marital status** ^b^			
* Unmarried*	54.0%	47.9%	40.7%
* Married*	34.4%	40.0%	46.4%
* Widowed*	11.2%	11.1%	9.7%
* Divorced/abandoned*	-	0.8%	3.1%
* Unknown*	0.5%	0.2%	0.2%
**Vital status patient’s parents**			
* Father deceased*	18.1%	13.8%	13.5%
* Mother deceased*	12.2%	13.5%	10.1%
* Both deceased*	40.3%	37.0%	30.9%
* Both alive*	29.4%	35.7%	45.6%
**Place of residence patient’s parents** ^c^			
* Amsterdam*	74.9%	62,5%	85,7%
* Other*	22.5%	21.0%	5.0%
* Unknown*	2.7%	16.5%	9.3%
**Children** ^d^			
* Without children*	67.3%	61.9%	57.4%
* With children*	32.7%	38.1%	42.6%

*Source*: Amsterdam Patient Records Database.

^a^For a reliable representation of the socioeconomic status of the patient population, this table refers only to those who were twelve years of age or older (the legal age for child labour since 1874). Children under the age of twelve can almost exclusively be categorised as unemployed within the category ‘no occupation’.

^b^With regard to marital status, this table only refers to those who were eighteen years of age or older. Patients under the age of eighteen can almost exclusively be categorised as ‘unmarried’, as a result of the legally established age of marriage (eighteen for men and sixteen for women).

^c^To determine the proximity of a family network, this includes only those patients whose place of residence is Amsterdam.

^d^Only patients older than eighteen years were included in this part of the analysis. In 1856 and 1876 none of the patients under the age of eighteen had children (as far as it can be determined from this source), and only a few of the patients under the age of eighteen had children in 1896.

## Sex

Almost equally as many women as men were admitted to the Binnengasthuis in the studied period. The hospital therefore did not seem to specifically serve or exclude patients of a particular sex. The fact that there was no evident gender distinction with regard to hospitalisation in the Binnengasthuis is contrary to the findings of Von Bueltzingsloewen, who stated that in most German hospitals at the time men were far more frequently hospitalised in comparison with women.^58^ Although Von Bueltzingsloewen does not elaborate on this, the different findings probably stem from differences in the population structure of the hospital’s surrounding region (service area), admission policies and the characteristics of the hospital—for example, in a military hospital logically more men than women were admitted. In contrast, in Amsterdam slightly more women inhabited the city during the whole time period: 139,263 women versus 120,610 men in 1856, and 259,927 women versus 234,297 men in 1896.^59^

In the various wards within the hospital, the ratio of men to women varied. In the surgical ward, where the wounded were treated for concussions, bone fractures, haemorrhages and other injuries, the majority of the patients were male. In all sample years about two-thirds of the patients admitted to this ward were men. Because of their presence in the public sphere, men were more likely to be ‘victims’ of workplace accidents, drunkenness and public disturbances. For example, in 1876, a 47-year-old workman was hospitalised, after he was ‘found on the *Houtgracht*, in inebriated state, under a burning cart’ and a 52-year-old carpenter was admitted to the hospital with a fractured neck after ‘falling from a plank, while working in a warehouse’. The fact that there is nevertheless a balance in the sex ratio is because there are also departments where only women are hospitalised, such as the gynaecology department and the maternity ward. In all other departments, men were overrepresented, for instance, in the hospital wards for the sick and those with venereal diseases.

## Age

With regard to the age of the patients in the Binnengasthuis, it seems that almost everyone—young and old—was admitted to the hospital. However, the classification into age categories also shows that the patient population was not evenly distributed across all age groups and was also subject to change over time. What can be established is that in all sample years the patients between 20 and 34 years of age (‘adults’) constituted the largest age group in the hospital, after all they also constituted the largest age group in Amsterdam.^60^ Furthermore, we see that initially mainly (young) adults were admitted to the hospital: nearly 75 per cent of all patients admitted in 1856 were between the ages of 14 and 49. Moreover, the sick and wounded in the youngest and oldest age groups seemed to avoid hospitalisation more often when just examining the numbers. Yet, no more than 10 per cent of the population was aged 65 or older during the entire study period. There were, however, many more infants and children among the Amsterdam population than there were in the Binnengasthuis. In the mid-19th century, infants and children up to the age of 13 made up about 30 per cent of the population, and in the hospital they represented only 12.5 per cent in 1856. This confirms earlier findings by Verdoorn that a significant number of infants was sick, and even died, without having received any medical treatment, especially in comparison to other age groups.^61^ Furthermore, the sharp increase of the group of patients between 0 and 13 years of age—children and infants—within the total patient population in the last quarter of the nineteenth century is particularly striking. A decline in infant mortality and a reduction of child mortality, starting around the 1880s, may underlie the increase in the proportion of the age group within the Amsterdam population and, subsequently, in the hospital.^62^ It is more plausible, however, that the growing awareness of children and infants as a special and unique group of patients in the Binnengasthuis led to an increase in admissions. For centuries, these sick children received no special attention and were placed here and there among the adults in the hospital wards. It was not until well into the nineteenth century that there was a growing awareness that paediatrics was a distinct medical field and separate wards were set up in the hospital for children (in 1871) and later also infants (in 1893).^63^ Although even at the end of the nineteenth century the (young) adults still constituted the largest age group, the number of children and elderly admitted did increase and over time the distribution of the patient population seems more diverse and a better reflection of the Amsterdam population.

## Place of Birth and Residence

During the second half of the nineteenth century the Binnengasthuis could be characterised as a public institution under the control of the city government, whose primary purpose was taking care of and healing the sick residents of the city of Amsterdam.^64^ Nevertheless, according to Hellinga, at the beginning of the nineteenth century half of the patients were not, or only for a short time, inhabitants of Amsterdam.^65^ As Table 4 shows, however, a vast majority of patients were residing, and born, in Amsterdam in the second half of the nineteenth century. The Binnengasthuis thus mainly served its own residents, next to some former residents of the city (who were born there but eventually had left the city).

Over time, the number of patients born outside Amsterdam increased and the number of patients born in Amsterdam decreased, which is probably a result of migration to the city during this period, which meant that a significant part of the inhabitants of Amsterdam originally came from other places. In 1896, comparatively more patients were born outside Amsterdam, but among them a relatively large share lived in Amsterdam: more than 85 per cent (compared to about 75 per cent in the earlier periods). Furthermore, with regard to the place of residence, an increasing number of patients lived in Amsterdam and a smaller proportion of all patients lived outside Amsterdam over time. The attraction of patients outside the city of Amsterdam therefore seemed to wane over time. Perhaps the sick and wounded were increasingly able to seek medical care in their own place of residence, as the number of new hospitals and the size of existing hospitals in the Netherlands rapidly increased during the nineteenth century.^66^

## Religion

In 1668, the city council established that no one could be refused admission to the Amsterdam Binnengasthuis based on his or her religious beliefs, which resulted in a wide diversity of religions and religious denominations within the patient population.^67^ As can be seen in Table 4, the vast majority of the patient population belonged to one of the various Christian denominations. Protestants—more than 70 per cent—and Catholics—about 28 per cent—formed the largest religious groups in the hospital, which is a fairly accurate reflection of the composition of the Amsterdam population at the time. Two in three residents were Protestant and just over 20 per cent of the population was Catholic. The absence of the Jewish population, which accounted for approximately 10 per cent of Amsterdam’s population throughout the nineteenth century, from the patient records is remarkable.^68^

Although the Jewish community formed a significant part of the Amsterdam population and the Binnengasthuis did not refuse sick and injured people based on their religious beliefs, a total of only 30 Jewish patients were admitted to the hospital in the sample years. Despite the public nature of the Binnengasthuis, it is possible that they avoided the hospital because it was not possible for them to continue the Jewish customs to which he or she was accustomed from home, for example, with regard to the food laws and the Sabbath. Moreover, they were probably admitted to the *Nederlands-Israëlitisch Ziekenhuis* (Dutch-Israelite Hospital). The hospital was founded as early as 1804, making it not only one of the first, but also the largest—though small in comparison to the Binnengasthuis—private hospitals in Amsterdam: growing from 446 patients in 1866 to over 1200 in 1896.^69^

## Socioeconomic Status

To determine the socioeconomic position of the patients, the Historical International Standard Classification of Occupations (HISCO) was used. In addition to the five classifications (HISCLASS_5), the category ‘no occupation’ has been added to the table. All patients for whom the original source explicitly stated ‘without occupation’ are included in this category. The socioeconomic status of these patients is not easy to determine. Perhaps some of them actually belonged to the lowest socioeconomic classes: ‘the poor who had no home, no clothes, and no food’.^70^ However, it is more likely that most were unemployed because the head of the household was—to some extent—able to support his family, since 62.1 per cent of female patients were classified in this category compared to 13.6 per cent of male patients. According to the labourers survey of 1887, the working population of Amsterdam consisted largely—about three quarters—of men.^71^ This finding is supported by the city’s occupational census data, which shows that in the second half of the nineteenth century, men constituted a significant majority of the workforce. For instance, in 1859, there were 75,820 men (73%) and 27,968 women (27%), and in 1889, there were 113,699 men (74%) and 39,855 women (26%). It is important to note that many women engaged in unpaid or unregistered work, and thus, were often not included or ‘under-registered’ in these surveys and counts.^72^

Women admitted to the hospital were mostly employed as servants, workers or seamstresses and consequently classified in the categories ‘skilled workers’ and ‘unskilled workers’. Most male patients were classified in the same socioeconomic classes based on their occupations as for example workmen, labourers, bakers, painters, shoemakers and carpenters. If we compare the situation in the hospital with that in the city of Amsterdam, based on the occupational data from the 1859 census, we find that among men the ‘elite’ and ‘lower middle classes’ are underrepresented and the working classes are overrepresented in the hospital. Among women, the elite and lower middle classes are again slightly underrepresented in the hospital. Furthermore, a remarkably large proportion of the women who were admitted to the hospital had jobs as (un)skilled workers compared to women in Amsterdam. In the hospital, only 62 percent of the women had no occupation, while in Amsterdam this was 78 percent. So it seems that mostly employed women found their way to the hospital.^73^

Moreover, it is noteworthy that over time, on the one hand, more and more patients ‘without occupation’ were admitted, and on the other hand—especially in the case of men—increasingly more patients from the upper and middle classes were admitted. The same development is reflected in the changes of the rental value categories: whereas the patients living in a house in the lowest rental value category (1) decreases over time, the other categories increase, in particular the share of the highest rental value (4). These results affirm earlier research on the patient population of hospitals during this period. The hospitalised population did not only consist of the so-called ‘industrious poor’ or ‘indigent’ (those whose existence depended on charity and poor relief, especially in times of sickness), but also many skilled workers and patients from the lower middle classes found their way to the Binnengasthuis.^74^ Moreover, contrary to Imhof’s findings, the sick and wounded from the middle and upper classes did not always succeed in avoiding hospitalisation, although they were mainly exceptions to the rule.^75^ Furthermore, as hospitals evolved over time, patient care at home became increasingly replaced by institutional hospital care, even among the more affluent population.^76^

## Family Network

In the second half of the nineteenth century, before there was a welfare state or social safety net, people were depending on relatives for support in times of need. Imhof observed that the hospital was above all intended for the sick and wounded who had no options other than hospitalisation, such as ‘the aged and the infirm without family’.^77^ Subsequently, Derosas and Munno concluded that those who could not rely on a family network, e.g. migrants, widows and orphans, were hospitalised more often than others.^78^  Table 4 includes several variables that can be related to the ‘family network’ of patients, such as the marital status, and the presence and proximity of their parents and children.

A significant proportion—overall 40 per cent—of the adult patients hospitalised in the Binnengasthuis were married. This is similar to the 31–36 per cent of the total population of Amsterdam being married during the studied time period.^79^ Only a relatively small proportion of the patient population was widowed, abandoned or divorced. Based on the assumption that married patients, as opposed to ‘singles’, could generally rely on their families, Derosas and Munno’s assumption that ‘those who resorted to hospital care [...] had little or no support from their families’ does not appear to be applicable to the Binnengashtuis.^80^ Furthermore, the group of unmarried patients provides a complex array of cases. On the one hand, most of them were probably still in a situation where they could count on the care and support of their family. On the other hand, this is not the case for everyone, especially those who were unmarried and admitted to the maternity ward or the ward for venereal diseases.^81^ The latter could count on little support and understanding within their family network due to stigmatisation. It is also worth noting that during the second half of the nineteenth century, increasingly more sick and injured people who were married chose to be admitted to the hospital. This indicates that despite being able to rely on their family network, people chose to be admitted. Subsequently, the proportion of patients without a familial safety net, such as widows and widowers, decreased over time.

The number of patients whose parents were deceased or lived outside Amsterdam and the number of patients without children, decreased during the course of the nineteenth century. This development is, however, partly a consequence of the changing age structure of the hospitalised population: the number of young patients admitted to the Binnengasthuis increased over time, with the result that the parents of patients were often still alive. It is therefore unclear if it can be argued that not only people with a small family network were admitted to the Binnengasthuis, and that this increased over time. Still, it is striking that over time, parents of patients increasingly did not live outside Amsterdam. This might suggest that patients could fall back on that network, but still chose to be hospitalised instead.

## Determinants of Mortality

**Fig. 2.**
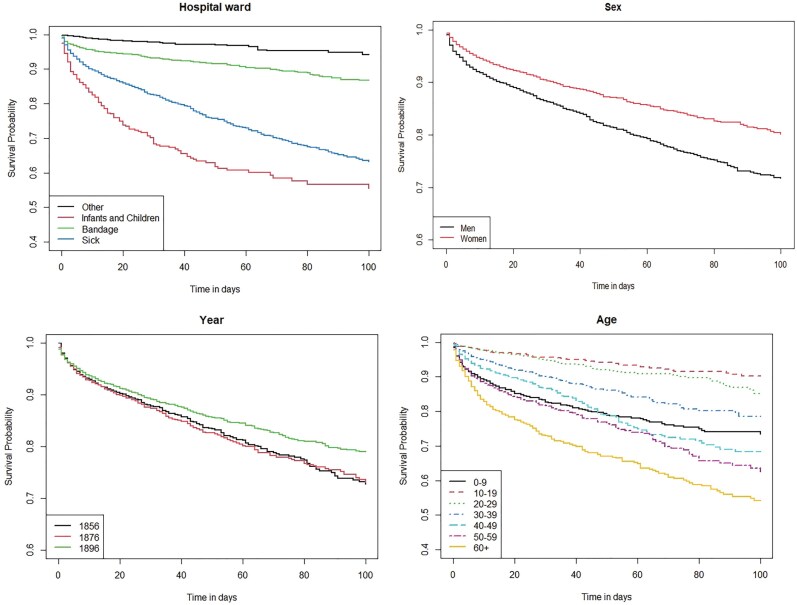
Kaplan–Meier survival curves by hospital ward, sex, year and age (0–100 days), 1856–1896. *Note*: Please note *y*-axis is not the same in each graph. *Source*: Amsterdam Patient Records Database

Having examined the institutional mortality patterns and patient composition, the focus of this section is on exploring the factors that contributed to the mortality risks of patients. Specifically, the aim is to investigate whether certain patient characteristics were associated with higher or lower mortality, and whether there were any indications of inequality within the hospital. This provides insight into the extent to which the hospital was serving different populations and whether it indicates if any disparities in the quality of care that patients received can be observed. To get a first understanding of what determined who had a higher risk of dying in the hospital, Figure 2 shows the survival until day 100 for several important determinants: hospital ward, year, sex and age. These are explored first because they represent the context for going to the hospital (ward and time period) and demographic factors (sex and age) that we expect to have an important influence on mortality risks and should be controlled for.

The determinants of mortality in hospitalised patients are largely dependent on the specific diseases from which they suffer, as we have shown in the first part of the results. While the recording of disease information is available only for the year 1896, the ward to which patients were admitted can provide meaningful insights into the types of diseases that were prevalent among admitted patients.^82^  Figure 2 demonstrates that wards in which infants and children were admitted faced the highest mortality risks, in particular after 30 days only 68 per cent survived. Thereafter, mortality risks were a bit lower, resulting in only 56 per cent of the admitted infants and children still being alive at day 100. This reflects the results of previous research on infant and child mortality in the general population, where in particular infant and young children have the highest mortality risks (for example, the IMR was around 200 in 1856 and declined to 164 in 1896).^83^ The patients admitted to the broad, and rather vague, category ‘Sick’ had the second highest mortality risks. Most patients with severe and deadly diseases were admitted to this hospital ward. In the first 10 days mortality risks were particularly high, with only 90 per cent surviving. From day 10 until 100 mortality risks were relatively similar, resulting in 63 per cent being still alive at the end of the period. As expected, the patients admitted to the wards ‘Bandage’ and ‘Other’ had much lower mortality risks, as most of their conditions were less severe and deadly. In turn, after 100 days 87 per cent of the patients of Bandage were still alive, and 94 per cent of the patients of Other. Important to note is that the difference between wards was statistically significant. As expected due to the overall decline in mortality in society, a considerable decrease in mortality in 1896 (79 per cent survived until day 100) compared to 1856 and 1876 (72 per cent survived until day 100) was observed. These differences in survival rates are statistically significant and suggest that the hospital became more efficient and effective in treating various diseases over time, or those admitted had less deadline diseases and injuries. Additionally, the opening of new wards that treated less fatal diseases, such as the wards for eye and skin diseases, likely contributed to this trend. Still, we have to take into account that 1896 was a year in which mortality was very low in the Binnengasthuis, compared to other years in the same decade.

When considering the impact of demographic factors, namely age and sex, patterns consistent with those observed in the general population were found. Firstly, the mortality rates of male patients were higher compared to female patients. By the end of the observation period (100 days), only 71 per cent of the male patients ‘survived’, compared to 80 per cent of the female patients. However, the magnitude of the difference between male and female mortality in the Binnengasthuis appears to be larger than expected and increased over time. This may in part be attributed due to the fact that more women were admitted to wards where mortality risks were much lower. Still, the differences observed between male and female mortality rates were not found to be statistically significant. Secondly, the older the patients admitted to the hospital, the higher the mortality risks, with only 54 per cent of the highest age group (60+) surviving until day 100, compared to 90 per cent and 84 per cent survival rates for patients in the age groups 10–19 and 20–29, respectively. Children under the age of 10, are the exception. They have mortality risks comparable to those observed in the 30–39 and 50–59 age groups, with a 73 per cent survival rate until day 100. These age differences were found to be statistically significant, reflecting that the most vulnerable groups are typically the very young and the elderly, also in the hospital.

To be able to go a step further and take into account multiple variables to estimate differences in mortality risks of our key determinants, in Table 5 Cox proportional hazard models are presented.^84^ Results confirm that mortality rates decreased over time, which is in line with broader societal trends and the finding that in 1896 mortality was very low in the Binnengasthuis. Results for age are also as expected, compared to the age group 14–34, younger and older patients had higher mortality risks. Despite religious differences between the groups analysed, there are no significant differences in mortality rates. The analysis on marital status demonstrates that except for higher mortality risks among widowed patients, no significant differences in mortality rates based on the marital status of the patients can be observed. Coming back to the argument in the previous section, the most likely explanation for the higher mortality risk among widowed patients is the lack of support from a family network, resulting in poorer health upon admission to the hospital and a higher likelihood of staying in the hospital when being deemed untreatable and consequently dying.

When turning to the indicators for social-economic status it seems that there are little indications for large differences between social groups or a social gradient. Rent value of the house a patient lived in—a first indicator of social-economic status—does show a lower mortality risk for patients in the third and fourth quartile, compared to ones in the first quartile. Only the differences of the patients in the third quartile of the rent value are statistically significant. When examining occupation as a second indicator for social-economic status, the results demonstrate that compared to unskilled workers, all other social-economic groups have higher mortality risks. This is contrary to previous research on the general population, which suggests that lower socioeconomic status leads to lower or equal survival chances. Two possible explanations may account for this discrepancy. Firstly, admission to the hospital wards could differ substantially between unskilled and skilled workers.^85^ Additional analyses, however, indicate that this mortality advantage persists even when controlling for hospital ward, and that unskilled and skilled workers were present in similar proportions across wards. Nevertheless, it is still possible that unskilled workers only sought hospitalisation for less severe ailments. Secondly, unskilled workers may have been more likely to leave the hospital before dying compared to skilled workers, and the lower and upper middle classes. This could introduce a selection bias, as we are unable to observe if patients died soon after leaving the hospital. Consequently, we cannot discount the possibility that unskilled workers experienced higher mortality rates outside of the hospital setting, which could offset the advantage observed within the hospital. In addition to occupation and rent value, another indicator of social status differences is the wealth classification based on the neighbourhood level. No significant differences in the general wealth level between neighbourhoods where patients resided are found. A lower mortality risk among patients whose neighbourhood of residence was not recorded can be observed. These findings suggest that this group likely included individuals who were not registered as Amsterdam residents, possibly indicating the ‘healthy migrant effect’, a phenomenon where migrants tend to be healthier than the local population. This can be attributed to the fact that individuals who choose to migrate are often healthier, as the process of migration itself can be physically and mentally demanding. As a result, migrants generally exhibit lower mortality rates and fewer health conditions compared to non-migrants.^86^ Alternatively, it is possible that these patients may have returned home before they died, which could also account for the lower mortality risk observed in this group.

## Conclusions

The main aim of our study was to explore the patient population and mortality in the Amsterdam Binnengasthuis in the second half of the nineteenth century. This was done to provide—for the first time—an insight into the development of hospitals and hospital care from a patient’s perspective in the Netherlands. Through analysing patient records, we could go beyond studying only institutional mortality, and examine the patient population and individual mortality risks in-depth.

Our examination of institutional mortality in the Amsterdam Binnengasthuis sheds light on its complex role as a healthcare institution in this period of medicalisation. The hospital was characterised by a high mortality rate (10–15 per cent), which distinguished it from other European hospitals at that time. However, the Binnengasthuis—unlike other case studies—was a large-scale and public institution, located in the centre of a rapidly growing city and characterised by a non-restrictive admission policy, which (as previous studies have already shown) had an evident impact on institutional mortality. However, the answer to the question whether the Binnengasthuis can be described as an actual ‘gateway to death’ remains complex, as Sigsworth pointed out: ‘if the hospital was in fact a so-called “gateway to death”, why were people willing to enter them at all?’. The Binnengasthuis remained a crucial site—no matter the outcome—for thousands of sick and injured people every year. They were prepared to enter the hospital, perhaps because they had no other options, but possibly also because the hospital was not considered a ‘gateway to death’ by its contemporaries. Moreover, circumstances and conditions in the hospital reflected the general state of public health in the city. Hence, the question arises: was the Binnengasthuis a gateway to death, or was Amsterdam as a whole a ‘death trap’, given the city’s deplorable living and working conditions, numerous outbreaks of infectious diseases and lack of sanitation?

While past perceptions characterised hospitals as healthcare institutions serving only the elderly, the poor and others from lower social strata, our analysis reveals an alternate historical reality, which is in line with the findings of previous studies.^87^ Our study demonstrates that a wide variety of patients—in terms of sex, age, origin, occupation, religion—was admitted to the Binnengasthuis, although the extent to which certain groups were represented varied. The patient population reflected the diversity of the Amsterdam population. In fact, a large proportion of all Amsterdam inhabitants have been admitted to the hospital at one time or another. Therefore it seems that the hospital was not necessarily avoided by the majority and only used by an unfortunate minority as a last resort, as has long been assumed by historians.

Our explorative examination of mortality risks among patients shows significant disparities across time, hospital wards, demographic factors (age and sex) and socioeconomic indicators (occupation). The most significant factors contributing to survival chances among patients were consistent with those observed in the general population, namely age and sex. This suggests the Amsterdam Binnengasthuis did not create or increase health inequalities. In short, as the context of the hospital (location, size, status, admission policy) influenced the institutional mortality rate, as Woodward observed, the social-demographic background and diagnosis affected a patient’s chances of survival and mortality.

In conclusion, our research offers a nuanced understanding of hospital care in nineteenth-century Amsterdam, revealing both challenges and triumphs in the journey from ‘gateways to death’ to medical centres attracting and serving an ever-growing part of society. By exploring the dynamics of mortality and patient populations in a hospital context, we contribute to a deeper understanding of historical healthcare systems and pave the way for future inquiries into healthcare inequalities and patient history. For example by investigating other hospitals (in the Netherlands), extending the analysis into the early twentieth century (due to the ongoing process of medicalisation) and take in account the lives of patients after hospitalisation.

## Footnotes

1 Anders Brändström and Göran Broström, ‘Life-Histories for Nineteenth-Century Swedish Hospital Patients: Chances of Survival’, *Journal of Family History*, 1989, 14, 195–209; Guenter B. Risse, *Medicalization: Hospitals Become Sites of Medical Care and Learning* (conference paper, 2004), 1–25; Renzo Derosas and Cristina Munno, ‘The Place to Heal and the Place to Die. Patients and Causes of Death in Nineteenth-Century Venice’, *Social History of Medicine*, 2022, 35, 1140–61; Isabelle von Bueltzingsloewen, ‘Pour une sociologie des populations hospitalisées: le recours à l’hôpital dans l’Allemagne du premier XIXe siècle’, *Annales de Démographie Historique*, 1994, 303–316; John Henderson, Peregrine Horden and Alessandro Pastore, eds, *The Impact of Hospitals 300–2000* (Bern: Peter Lang, 2007), 15–56.

2 Johannes A. Verdoorn, *Het gezondheidswezen te Amsterdam in de 19*^*e*^  *eeuw* (Nijmegen: SUN, 1981), 131–50; Frank Tang and Margriet Wigard, *Amsterdamse gasthuizen vanaf de Middeleeuwen* (Amsterdam: Amsterdam University Press, 1994), 23, 43–50; Gerhardus Hellinga, *Geschiedenis van het St. Pieters- of Binnengasthuis* (Amsterdam: Stadsdrukkerij Amsterdam, 1930), 70–5; Daniël de Moulin, ed, *Vier eeuwen Amsterdams Binnengasthuis* (Wormer: Inmerc, 1981), 4–19.

3 Moulin, *Vier eeuwen Amsterdams Binnengasthuis*, 83.

4 Christianus Johannes Nieuwenhuys, *Proeve eener geneeskundige plaatsbeschrijving (topographie) der stad Amsterdam III* (Amsterdam: Johannes van der Hey, 1820), 158.

5 Derosas and Munno, ‘The Place to Heal’, 1143.

6 Hellinga, *Geschiedenis van het*, 116; Tang and Wigard, *Amsterdamse gasthuizen*, 8.

7 Grosvenor T. Griffith, *Population Problems of the Age of Malthus* (Cambridge: Cambridge University Press, 1926); Steven Cherry, ‘The Role of a Provincial Hospital: The Norfolk and Norwich Hospital, 1771-1880’, *Population Studies*, 1972, 26, 291–306.

8 Thomas McKeown, *The Modern Rise of Population* (London: Edward Arnold, 1976); Thomas McKeown, *The Role of Medicine: Dream, Mirage or Nemesis?* (London: Nuffield Provincial Hospitals Trust, 1976).

9 Thomas McKeown and R. G. Brown, ‘Medical Evidence Related to English Population Changes in the Eighteenth Century’, *Population Studies*, 1955, 9, 119–41, 119–24.

10 Phyllis Deane, *The First Industrial Revolution* (Cambridge: Cambridge University Press, 1965); Peter Mathias, *The First Industrial Nation. An Economic History of Britain 1700-1914* (London and New York: Routledge, 1969), 189.

11 McKeown and Brown, ‘Medical Evidence’, 125.

12 Henderson, Horden and Pastore, *The Impact of Hospitals,* 26.

13 Brändström and Broström, ‘Life-Histories’, 198–9; Derosas and Munno, ‘The Place to Heal’, 1146; Risse, *Medicalization*, 10.

14 Eric M. Sigsworth, ‘Gateways to Death? Medicine, Hospitals and Mortality, 1700–1850’, in Peter Mathias, ed, *Science and Society 1600-1900* (Cambridge: Cambridge University Press, 1972), 97–110.

15 John Woodward, *To Do the Sick No Harm. A Study of the British Voluntary Hospital System to 1875* (London: Routledge, 1974).

16 Brändström and Broström, ‘Life-Histories’, 195–209.

17 These observations are also related to the (relatively high or low) mortality risks of certain diseases in general.

18 Roy Porter, ‘The Patient’s View: Doing Medical History from Below’, *Theory and Society*, 1985, 14, 175–98; Henderson, Horden and Pastore, *The Impact of Hospitals,* 35–46; Brändström and Brostrom, ‘Life-Histories’, 195–6; Arthur E. Imhof, ‘The Hospital in the 18th century; For whom? The Charite Hospital in Berlin, the Navy Hospital in Copenhagen, the Kongsberg Hospital in Norway’, *Journal of Social History*, 1977, 10, 448–70, 455.

19 Imhof, ‘The Hospital’, 448–70.

20 Brändström and Broström, ‘Life-Histories’, 195–209; Von Bueltzingsloewen, ‘Pour une sociologie’, 303–16.

21 Derosas and Munno, ‘The Place to Heal’, 1155.

22 Tang and Wigard, *Amsterdamse gasthuizen*, 7–8.

23
*Ibid*., 27; Hellinga, *Geschiedenis van het*, 22.

24 Tang and Wigard, *Amsterdamse gasthuizen*, 49; Moulin, *Vier eeuwen Amsterdams Binnengasthuis*, 17–8.

25 Tang and Wigard, *Amsterdamse gasthuizen*, 43; Annet Mooij, *De polsslag van de stad: 350 jaar academische geneeskunde in Amsterdam* (Amsterdam-Antwerpen: De Arbeiderspers, 1999), 157.

26 Arie Querido, *Godshuizen en gasthuizen* (Amsterdam: Em. Querido’s Uitgevermaatschappij, 1960), 34.

27 Moulin, *Vier eeuwen Amsterdams Binnengasthuis*, 19.

28 Verdoorn, *Het gezondheidswezen te Amsterdam*, 143.

29 Hellinga, *Geschiedenis van het*, 70–5.

30 Moulin, *Vier eeuwen Amsterdams Binnengasthuis*, 19; Verdoorn, *Het gezondheidswezen te Amsterdam*, 135.

31 Moulin, *Vier eeuwen Amsterdams Binnengasthuis*, 20–1; Tang and Wigard, *Amsterdamse gasthuizen*, 52.

32 Hellinga, *Geschiedenis van het*, 51–6; Tang and Wigard, *Amsterdamse gasthuizen*, 53–4.

33 Moulin, *Vier eeuwen Amsterdams Binnengasthuis*, 22–3; Tang and Wigard, *Amsterdamse gasthuizen*, 56–7.

34 Moulin, *Vier eeuwen Amsterdams Binnengasthuis*, 25–6.

35 Mooij, *De polsslag van de stad*, 202–3; Moulin, *Vier eeuwen Amsterdams Binnengasthuis*, 28–9.

36 Hellinga, *Geschiedenis van het*, 78–9; Moulin, *Vier eeuwen Amsterdams Binnengasthuis*, 39-42; Tang and Wigard, *Amsterdamse gasthuizen*, 63–5.

37 Mooij, *De polsslag van de stad*, 225–26; Verdoorn, *Het gezondheidswezen te Amsterdam*, 135–36.

38 Stadsarchief Amsterdam (Amsterdam City Archives), 5268: Archief van de Gemeenteziekenhuizen, inventory number 2511-2610.

39 Stadsarchief Amsterdam (Amsterdam City Archives), 15030: Inventaris van de Collectie Stadsarchief Amsterdam: bibliotheek, inventory number 133253-133302.

40 Angélique Janssens, Owen Lammertink, Tim Riswick, Sanne Muurling, Ernst Kuiper, Minke Twijnstra, **Amsterdam Cause-of-death Database*, *1854–1940* *(2023), version 1.0, unpublished dataset Radboud Group for Historical Demography and Family History, Radboud University, The Netherlands.

41 Angélique Janssens, ‘Constructing SHiP and an International Historical Coding System for Causes of Death’, *Historical Life Course Studies*, 2021, 10, 64–70.

42 Alice Reid *et al*., ‘“A Confession of Ignorance”: Deaths from Old Age and Deciphering Cause-of-Death Statistics in Scotland, 1855–1949’, *The History of the Family*, 2015, 20, 320–44, 324.

43 Owen Lammertink, *De opkomst van het moderne ziektepatroon? Doodsoorzaken, degeneratieve aandoeningen en sociale ongelijkheid in Amsterdam, 1854-1926* (Nijmegen, 2023), 133–37.

44 Sanne Muurling, Tim Riswick & Katalin Buzasi, ‘The Last Nationwide Smallpox Epidemic in the Netherlands: Infectious Disease and Social Inequalities in Amsterdam, 1870-1872’, **Social Science History*, * 2023, 47:2, 189–216. Tim Riswick , Sanne Muurling & Katalin Buzasi, ‘Exploring the mortality advantages and disadvantages of Jewish neighborhoods in nineteenth century Amsterdam’, **Demographic Research*, * 2022, 46:25, 723–736.

45 We look at the first 100 days of hospitalisation for a first explorative view on survival chances among patients. This is our starting point here because the vast majority of patients (93.2 per cent) leave the hospital within this timeframe. Only a small and exceptional proportion of patients stayed for a longer period of time in the hospital. Patients staying in the hospital for a longer period of time probably had different underlying reasons for staying in the hospital (care instead of cure).

46 Göran Broström, *Event History Analysis with R* (Boca Raton: CRC Press, 2012).

47 We look at the first 10 days of hospitalisation because most patients die in the first days after admission (about half of the patients die before day 10).

48 This also indicates that the effects are not constant over time if you study the full 100 days after hospitalisation.

49 Stadsarchief Amsterdam (Amsterdam City Archives), 15030: Inventaris van de Collectie Stadsarchief Amsterdam: bibliotheek, inventory number 133254, 20.

50 Stadsarchief Amsterdam (Amsterdam City Archives), 15030: Inventaris van de Collectie Stadsarchief Amsterdam: bibliotheek, inventory number 133279, 102–3.

51 Cherry, ‘The Role of a Provincial Hospital’, 300; Risse, *Medicalization*, 14.

52 McKeown and Brown, ‘Medical Evidence’, 119–20.

53 Brändström and Broström, ‘Life-Histories’, 197.

54 Cherry, ‘The Role of a Provincial Hospital’, 295–96.

55 Woodward, *To Do the Sick No Harm*, 46–9, 155–56.

56 Verdoorn, *Het gezondheidswezen te Amsterdam*, 55–6.

57 Reid *et al*., ‘A Confession of Ignorance’, 340.

58 Von Bueltzingsloewen, ‘Pour une sociologie’, 307.

59 Stadsarchief Amsterdam (Amsterdam City Archives), 15030: Inventaris van de Collectie Stadsarchief Amsterdam: bibliotheek, inventory number 133258 and 133298.

60 Verdoorn, *Het gezondheidswezen te Amsterdam,* 30.

61
*Ibid*., 60; Willibrord J. M. J. Rutten, ‘Ongelijke behandeling binnen het gezin: een onderzoek naar de leeftijdsverschillen in de kans op geneeskundige hulp in Nederland (ca. 1870-1900)’, *A.A.G. Bijdragen,* 1986, 28, 245–65; Willibrord Rutten, ‘An Outline of Socio-Medical Care in the Netherlands, 19th and early 20th Century’, in Theo Engelen, John R. Shepherd and Yang Wen-shan, eds, *Death at the Opposite Ends of the Eurasian Continent. Mortality Trends in Taiwan and the Netherlands 1850–1945* (Amsterdam: Aksant Amsterdam, 2011).

62 Verdoorn, *Het gezondheidswezen te Amsterdam*, 57, 62.

63 Moulin, *Vier eeuwen Amsterdams Binnengasthuis*, 37–8; Tang and Wigard, *Amsterdamse gasthuizen*, 62.

64 The origin of this established role of the Binnengasthuis can be traced back to 1686, when the city council decided that the hospital could no longer fulfil its function as vreemdelingenhuis (shelter for foreigners), because the limited available resources were to be used for the benefit of the city’s own residents. See Tang and Wigard, *Amsterdamse gasthuizen*, 27.

65 Hellinga, *Geschiedenis van het*, 22.

66 Lia de Vos, *Kwaliteit, disciplinering en sturing. Een historisch sociologisch onderzoek naar de vormgeving van kwaliteit van zorg in ziekenhuizen* (Rotterdam, 2014), 65.

67 Moulin, *Vier eeuwen Amsterdams Binnengasthuis,* 82.

68 Verdoorn, *Het gezondheidswezen*, 33–5.

69
*Ibid*., 137.

70 Louis Pappenheim, *Handbuch der Sanitäts-Polizei* (Berlin: Hirschwald, 1859).

71 Jacques Giele, *De arbeidsenquête van 1887. Deel 1: Amsterdam* (Nijmegen: Uitgeverij Link, 1981).

72 Volkstellingen: beroepen (censuses: occupations) of Amsterdam 1859 & 1889, accessibele via: <http://www.volkstellingen.nl/>

73 see Appendix

74 Brändström and Broström, ‘Life-Histories’, 198–99; Von Bueltzingsloewen, ‘Pour une sociologie’, 310–11, 316.

75 Imhof, ‘The Hospital’, 451.

76 Derosas and Munno, ‘The Place to Heal’.

77 Imhof, ‘The Hospital’, 451.

78 Derosas and Munno, ‘The Place to Heal’, 3.

79 Het Bureau van Statistiek der Gemeente, *Statistisch Jaarboek der Gemeente Amsterdam* (Amsterdam, 1896), 21.

80 Derosas and Munno, ‘The Place to Heal’, 10.

81 Tim Riswick, Mayra Murkens & Nadeche Diepgrond, ‘Moeders in nood: patiënten op de kraam- en gynaecologieafdelingen van het Amsterdamse Binnen- en Buitengasthuis, 1856–1896’, in: Annemie Leemans & Cornelis van Tilburg (eds.), *Cahiers Geschiedenis van de Geneeskunde en Gezondheidszorg 18–Geschiedenis van de geboorteregeling en familieplanning* (Antwerpen, 2024).

82 In the first Kaplan–Meier curve four categories are visualised: Sick (‘Sick’), Bandage (‘Child Bandage’ and ‘Bandage’), Infants and Children (‘Infants’, ‘Children Sick’, ‘Children’) and Other (‘Gynaecology’, ‘Skin disease’, ‘Child skin disease’, ‘Eye disease’, ‘Syphilis’, ‘Mental sick’, ‘Maternity’).

83 Angélique Janssens & Tim Riswick, ‘What was killing babies in Amsterdam? A study of infant mortality patterns using individual level cause-of-death data, 1856–1904’, *Historical Life Course Studies* 13, 2023, 235–264.

84 As a robustness check the same model was applied to the sick ward only, and results were very similar: the direction of the effect was in all cases the same. The following variables were, however, not statistically significant: period, rent value and age group 5–13. Models for the (other) separate wards are not presented because the number of events becomes too low for reliable results.

85 For instance, if unskilled workers were primarily admitted for minor ailments, such as bandaging, whereas skilled workers were more likely to require treatment for serious illnesses, this could explain the mortality advantage observed in the former group.

86 Rick J. Mourits and Paul Puschmann, ‘Exploring Familial Factors in the Migrant Mortality Advantage Among Domestic Migrants in Later Life: Zeeland, the Netherlands, 1812–1962’, *SSM - Population Health*, 2023, 22.

87 Brändström and Broström, ‘Life-Histories’, 195–209; Von Bueltzingsloewen, ‘Pour une sociologie’, 303–16.

## Notes

Nadeche Diepgrond is currently a PhD Candidate at Radboud Group for Historical Demography and Family History, Radboud University, the Netherlands. She is interested in medical history and historical demography, specifically the history of hospitals and patients. Her dissertation examines the ‘health transition’ within Dutch hospitals and inequalities among hospital patients in the long 19th century.

Tim Riswick is currently an assistant professor of historical demography at the Radboud Group for Historical Demography and Family History, Radboud University, the Netherlands. His expertise focuses specifically on the impact of specific determinants on (cause-specific) mortality risks, and (comparative) family history research on East Asia and Western Europe in general.

## Appendix

**Table:**

Occupational group	Men		Women	
	Hospital	Amsterdam	Hospital	Amsterdam
Elite	0.40%	1.81%	-	0.08%
Lower middle class	7.90%	20.97%	1.10%	3.01%
Skilled workers	40.80%	29.32%	4.50%	3.31%
Farmers and fishermen	0.40%	0.59%	-	0.01%
Unskilled workers	34.70%	29.56%	32.00%	15.19%
Occupation unknown	2.20%	-	0.30%	-
No occupation	13.60%	17.73%	62.10%	78.41%

Source: Volkstellingen: beroepen (censuses: occupations) of Amsterdam 1859, accessible via http://www.volkstellingen.nl/

